# Histological Features of Kidney Allograft Biopsies According to Metabolic Acidosis Status: A Biopsy-Based Single-Center Observational Study

**DOI:** 10.3390/life16010097

**Published:** 2026-01-09

**Authors:** Lucian Siriteanu, Andreea Simona Covic, Călin Namolovan, Mihai Onofriescu, Simona Mihaela Hogaș, Luminița Voroneanu, Irina-Draga Căruntu, Mehmet Kanbay, Adrian Covic

**Affiliations:** 1 Department of Nephrology, Grigore T. Popa University of Medicine and Pharmacy, 700115 Iași, Romania; siriteanulucian@gmail.com (L.S.); mihai.onofriescu@gmail.com (M.O.); simonamihaelahogas@gmail.com (S.M.H.); lumivoro@yahoo.com (L.V.); irinadragacaruntu@gmail.com (I.-D.C.); adrianccovic@gmail.com (A.C.); 2 Clinical Hospital “Dr. C. I. Parhon”, 700503 Iași, Romania; calinnamolovan67@gmail.com; 3 Department of Medicine, Division of Nephrology, Koc University School of Medicine, Istanbul 34450, Turkey; mkanbay@ku.edu.tr

**Keywords:** kidney transplantation, metabolic acidosis, renal allograft biopsy, histopathology, Banff classification, interstitial fibrosis and tubular atrophy

## Abstract

Metabolic acidosis is common after kidney transplantation and has been linked to adverse renal outcomes. However, its relationship with histological injury in kidney allografts remains poorly characterized. We aimed to explore the association between metabolic acidosis and histopathological features in kidney allograft biopsies. This single-center, cross-sectional observational study included 63 adult kidney transplant recipients who underwent clinically indicated allograft biopsies. Metabolic acidosis was defined as a serum bicarbonate level < 22 mmol/L at the time of biopsy. Histological lesions were assessed according to the Banff classification. Lesion severity was evaluated using descriptive statistics, nonparametric comparisons, ordinal logistic regression, and multivariable logistic regression models adjusted for renal function, proteinuria, and time from transplantation. Sensitivity analyses additionally adjusted for hemoglobin and donor-related variables. Patients with metabolic acidosis exhibited numerically higher severity scores for both acute inflammatory lesions and chronic histological changes, including total inflammation and interstitial fibrosis/tubular atrophy (IFTA). Across ordinal analyses and multivariable regression models, consistent directional trends toward a greater histological injury burden were observed among acidotic patients; however, none of these associations reached statistical significance, and confidence intervals were wide. Sensitivity analyses yielded directionally consistent effect estimates. In this biopsy-based analysis, metabolic acidosis showed consistent directional trends toward a higher burden of inflammatory and chronic histological lesions, although these findings did not reach statistical significance.

## 1. Introduction

Metabolic acidosis is common after kidney transplantation, with reported prevalence rates between 12% and 58% in the first year post-transplant [1,2,3,4]. Renal function trajectories after transplantation are highly heterogeneous. The predominant mechanism in the pathogenesis of metabolic acidosis is distal tubular dysfunction, characterized by impaired acid excretion, largely driven by immunological injury and ischemic processes affecting the renal allograft [5,6]. In kidney transplant recipients, metabolic acidosis may occur independently of overt renal function decline, and several transplant-specific factors are implicated, including immunosuppressive regimens—particularly calcineurin inhibitors and corticosteroids—as well as metabolic disturbances such as anemia and functional alterations of tubular acid–base transport proteins [4,7,8].

Metabolic acidosis in kidney transplant recipients has been consistently associated with adverse graft-related and systemic outcomes. Observational studies indicate that lower serum bicarbonate levels are independently linked to an increased risk of graft failure and all-cause mortality, even after adjustment for baseline renal function [2,9,10,11]. Beyond its impact on graft survival, metabolic acidosis contributes to multiple systemic complications, including bone disease, muscle wasting, anemia, heightened cardiovascular risk, and higher hospitalization rates [10,12,13,14].

In kidney transplant recipients, metabolic acidosis has been associated with impaired graft function and worse long-term outcomes. However, most available evidence relies on functional endpoints, such as estimated glomerular filtration rate [11,15], while data linking metabolic acidosis to direct histopathological allograft injury are scarce. Experimental models have suggested an association between metabolic acidosis and more severe histological injury, characterized by maladaptive repair processes leading to interstitial fibrosis and tubular atrophy. However, biopsy-based studies specifically assessing the relationship between metabolic acidosis and inflammatory or fibrotic allograft lesions remain limited [13,14,16].

Therefore, the aim of the present study was to investigate the association between metabolic acidosis and histological allograft injury in kidney transplant recipients undergoing clinically indicated renal biopsy. We specifically evaluated the relationship between metabolic acidosis and both inflammatory and chronic histological lesions.

## 2. Materials and Methods

### 2.1. Study Design

This study was conducted within a retrospective, single-center observational cohort of kidney transplant recipients (KTRs) followed at our tertiary transplant center. Although transplant recipients are routinely followed longitudinally as part of standard post-transplant care, the present analysis is based on a cross-sectional baseline evaluation, using data retrieved retrospectively from institutional medical records.

### 2.2. Study Population

We included adult kidney transplant recipients (≥18 years) with a functioning renal allograft for at least 3 months post-transplant who were attending routine outpatient follow-up during the study period and had complete baseline clinical and laboratory data, including serum bicarbonate. Patients evaluated between January 2023 and December 2023 were eligible. All recipients are routinely followed according to a standardized transplant care protocol; however, this report focuses exclusively on the cross-sectional baseline assessment.

Only patients with complete clinical, laboratory, and histological data at baseline were included; no imputation for missing data was performed. Patients with graft loss or return to dialysis, clinically overt biopsy-proven acute rejection, acute kidney injury requiring urgent intervention, active infection, or hospitalization within the preceding 4 weeks were excluded, as were those with missing key clinical or laboratory variables.

The primary aim of the parent cohort was to characterize the clinical phenotype of metabolic acidosis in kidney transplant recipients and to compare patients with and without metabolic acidosis. For this manuscript, we derived a biopsy sub-cohort from the parent population, consisting of patients who underwent clinically indicated renal allograft biopsy as part of the same baseline clinical evaluation and had complete contemporaneous clinical, biochemical, and histological data available.

Only renal allograft biopsies performed at baseline or within a short contemporaneous time window related to the same clinical episode were included; biopsies obtained at unrelated time points were excluded.

Evaluating metabolic acidosis status and renal histopathology at the same baseline time point enables a direct cross-sectional assessment of whether metabolic acidosis is associated with a distinct histological phenotype.

### 2.3. Biopsy Indication, Procedure and Histopathological Analysis

Renal allograft biopsies were performed exclusively on clinical indication, in accordance with routine clinical practice. Indications included: graft dysfunction (a rise in serum creatinine from previously stable values), new or worsening proteinuria, or other clinical or immunological suspicion of allograft injury. No protocol biopsies were performed. Importantly, biopsy indication reflected clinical suspicion rather than necessarily clinically confirmed acute rejection; therefore, histological examination could reveal lesions that were not clinically evident at baseline.

All kidney biopsies were performed at a single center using ultrasound-guided percutaneous techniques in accordance with standardized institutional protocols. Biopsy specimens were independently evaluated by two experienced renal pathologists. Pathologists were blinded to metabolic acidosis status, although routine clinical information was available in accordance with standard diagnostic practice. Histopathological assessment was primarily based on light microscopy, and immunofluorescence staining for C4d was performed in selected cases when available. Only biopsies providing sufficient cortical tissue to allow reliable Banff scoring were considered adequate for inclusion. Specimens deemed non-diagnostic were excluded.

Renal transplant biopsies were assessed according to the Banff Classification for Renal Allograft Pathology (2022), an internationally accepted expert consensus classification system for renal allograft pathology [17]. Acute T cell–mediated rejection (TCMR) and active antibody-mediated rejection (AMR) were defined by the presence of active inflammatory lesions, including interstitial inflammation (i), tubulitis (t), intimal arteritis (v), glomerulitis (g), peritubular capillaritis (ptc), and C4d deposition, where available. Chronic allograft injury was defined by chronic structural damage, including interstitial fibrosis and tubular atrophy (ci and ct), transplant glomerulopathy (cg), and chronic vascular changes. In addition, chronic active TCMR was identified according to Banff criteria using total cortical inflammation (ti) and inflammation within areas of IFTA (i-IFTA), with scores ≥ 2 required to meet diagnostic thresholds.

All individual histological lesions were semi-quantitatively scored from 0 (absent) to 3 (severe), using the exact morphological definitions specified in the Banff guideline. Other histopathological findings were interpreted in accordance with internationally accepted standards. In the event of a discrepancy, the final diagnosis was reached by consensus.

### 2.4. Definition of Metabolic Acidosis

Metabolic acidosis was defined as a serum bicarbonate concentration < 22 mmol/L at the time of the baseline clinical evaluation corresponding to the renal allograft biopsy. Classification of metabolic acidosis was based solely on this contemporaneous bicarbonate measurement, and values obtained at other time points were not used for exposure assignment.

### 2.5. Statistical Analysis

Continuous variables were tested for distribution and are presented as medians with interquartile ranges (IQRs). Comparisons between groups were performed using the Mann–Whitney U test for continuous variables and Fisher’s exact test for categorical variables, as appropriate. Histological scores were analyzed both as ordinal variables and as binary variables, defined as the presence of lesions (score ≥ 1). Ordinal logistic regression models were used to explore the association between metabolic acidosis and the severity of individual Banff histological lesions, treated as ordered categorical variables. Ordinal regression analyses focused on the main Banff histological lesions with sufficient variability and prevalence for reliable modeling. Less frequent lesions and those with highly skewed distributions were not included in ordinal models because of limited statistical power and model instability. To evaluate the association between metabolic acidosis and histological outcomes, multivariable logistic regression models were fitted with (i) total inflammation and (ii) interstitial fibrosis and tubular atrophy (IFTA) as binary outcomes. Metabolic acidosis was included as the primary exposure variable. Models were adjusted for baseline estimated glomerular filtration rate (eGFR) and proteinuria (urine protein-to-creatinine ratio). Given the limited sample size of the biopsy subgroup, multivariable regression models were intentionally kept parsimonious to minimize the risk of overfitting and model instability. Covariate selection was guided by clinical relevance and biological plausibility. Baseline eGFR and proteinuria were selected a priori as the most important confounders because they reflect renal function and injury severity and are closely linked to both metabolic acidosis and histological damage.

Other clinically relevant variables, including donor characteristics and hemoglobin, were not included in the primary multivariable models because of concerns about collinearity, limited statistical power, and potential overadjustment in a small cohort. To address potential residual confounding, sensitivity analyses were performed by additionally adjusting the multivariable models for hemoglobin levels and for donor-related variables (donor age and donor type). These analyses were undertaken to assess the robustness of the observed associations under alternative, but biologically plausible, modeling assumptions. Results are reported as odds ratios (ORs) with 95% confidence intervals (CIs). All statistical analyses were performed using R software (version 4.5.1; R Foundation for Statistical Computing, Vienna, Austria). A two-sided *p*-value < 0.05 was considered statistically significant.

### 2.6. Ethical Consideration

The study was conducted in accordance with the Declaration of Helsinki and approved by the Institutional Ethics Committee of “Grigore T. Popa” University of Medicine and Pharmacy, Iași (approval no. 532/5 February 2025).

## 3. Results

### 3.1. Study Population and Baseline Characteristics

The present study included 63 kidney transplant recipients who underwent clinically indicated renal allograft biopsy at baseline and had complete histological data available. At the time of biopsy, 41 patients (65.1%) met the criteria for metabolic acidosis, defined as a serum bicarbonate level < 22 mmol/L.

Baseline demographic, clinical, transplant-related, immunological, and laboratory characteristics of the biopsied cohort, stratified by metabolic acidosis status, are shown in Table 1.

Overall, the cohort consisted of relatively young kidney transplant recipients (median age 43 years), predominantly male, with a median time from transplantation of 24 months. Donor characteristics (donor type and donor age) and transplant-related factors (induction therapy, delayed graft function, prior sensitization, and maintenance immunosuppression) were generally comparable between patients with and without metabolic acidosis, with no statistically significant differences.

Patients with metabolic acidosis had a less favorable graft profile, with lower eGFR, higher creatinine, and higher proteinuria, along with lower hemoglobin, more frequent anemia, and higher CRP. These observations are descriptive and should be interpreted cautiously, given the limited sample size and cross-sectional design. Blood pressure, comorbidity burden, and cardiovascular risk factors were generally similar between groups.

### 3.2. Histological Findings

#### 3.2.1. Active Histological Lesions

**Figure 1:**
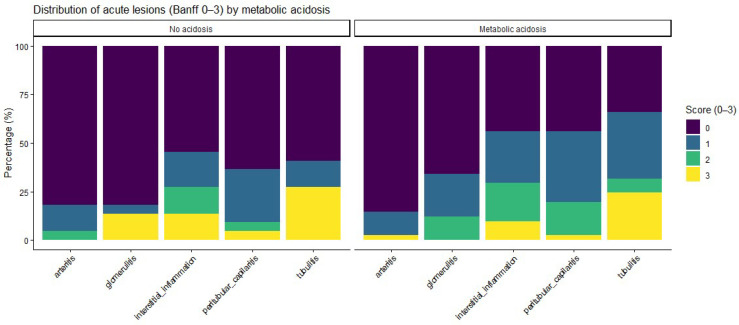
Heatmap illustrating the distribution of acute histological lesion severity according to metabolic acidosis status. For each Banff lesion, rows represent severity scores (0–3), and color intensity indicates the proportion of patients within each acidosis group exhibiting the corresponding score. Observed differences between groups are descriptive and did not reach statistical significance. No acidosis, n = 22; Metabolic acidosis, n = 41.

Acute histological lesions, assessed according to the Banff classification, are summarized in Table 2 and Figure 1. Patients with metabolic acidosis had numerically higher severity scores for inflammatory lesions, including glomerulitis, peritubular capillaritis, interstitial inflammation, and tubulitis, than patients without acidosis, whereas no relevant differences were observed for arteritis. When analyzed as ordinal severity scores, median [IQR] Banff grades were consistently higher among acidotic patients; however, Wilcoxon rank-sum testing did not show statistically significant differences between groups (Supplementary Table S1).

#### 3.2.2. Chronic Histological Lesions

**Figure 2:**
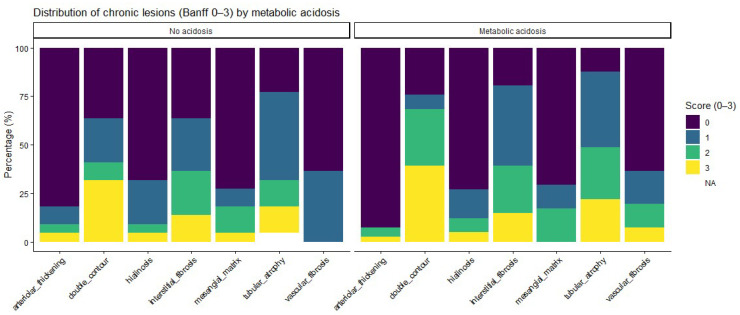
Heatmap illustrating the distribution of chronic histological lesion severity according to metabolic acidosis status. For each Banff lesion, rows represent severity scores (0–3), and color intensity indicates the proportion of patients within each acidosis group exhibiting the corresponding score. Observed differences between groups are descriptive and did not reach statistical significance. No acidosis, n = 22; Metabolic acidosis, n = 41.

Regarding chronic histological lesions, patients with metabolic acidosis had numerically higher scores for interstitial fibrosis and tubular atrophy, as well as for other chronic changes. Detailed distributions are provided in Table 3 and Figure 2. Ordinal analyses of chronic lesion scores also showed a consistent trend toward a higher chronic damage burden in acidotic patients, although none of these differences reached statistical significance (Supplementary Table S1).

#### 3.2.3. Total Inflammation and IFTA

Ordinal distributions of total cortical inflammation and IFTA (scores 0–3) suggested a trend toward higher grades among acidotic patients; however, these patterns were not statistically significant in Wilcoxon rank-sum tests (Table 4).

**Figure 3:**
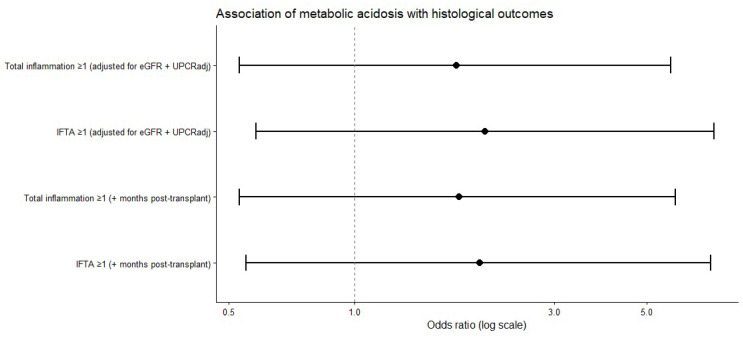
Forest plot showing adjusted odds ratios and 95% confidence intervals for selected histological outcomes according to metabolic acidosis status. Models were adjusted for baseline renal function, proteinuria, and time from transplantation. None of the associations reached statistical significance.

When histological lesions were analyzed as composite binary outcomes, the prevalence of both total inflammation (score ≥ 1) and interstitial fibrosis and tubular atrophy (IFTA, score ≥ 1) was higher among patients with metabolic acidosis than among those without acidosis. In multivariable logistic regression analyses adjusted for renal function, proteinuria, and time from transplantation, metabolic acidosis was consistently associated with higher odds of both total inflammation and IFTA, although these associations did not reach statistical significance (Table 5, Figure 3).

Sensitivity analyses, including additional adjustment for hemoglobin and donor-related variables, yielded directionally consistent effect estimates for metabolic acidosis across both total inflammation and IFTA (Supplementary Table S2).

#### 3.2.4. Rejection Phenotypes

**Figure 4:**
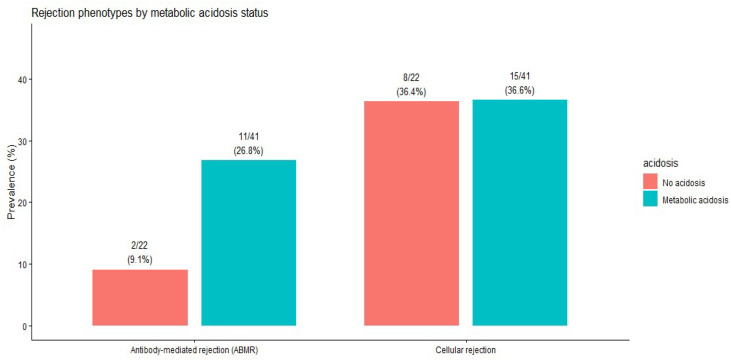
Distribution of rejection phenotypes according to metabolic acidosis status. Bars represent the proportion of patients in each rejection category. Differences between groups are descriptive and were not statistically significant.

The prevalence of cellular and antibody-mediated rejection did not differ significantly by metabolic acidosis status (Table 6). The distribution of rejection phenotypes by acidosis status is shown in Figure 4.

## 4. Discussion

In this biopsy-based, cross-sectional analysis of kidney transplant recipients, we observed consistent directional trends linking metabolic acidosis with a greater burden of both acute inflammatory and chronic histological lesions. Across descriptive analyses, ordinal comparisons, and multivariable regression models, patients with metabolic acidosis tended to have higher severity scores for inflammatory lesions and greater chronic damage, as reflected by total inflammation and IFTA. Importantly, none of these associations reached statistical significance after adjustment, underscoring the exploratory and hypothesis-generating nature of the present findings.

When individual Banff lesion scores were analyzed as ordinal severity scales, acidotic patients had numerically higher grades for key acute inflammatory lesions—including glomerulitis, peritubular capillaritis, interstitial inflammation, and tubulitis—as well as for chronic structural changes such as interstitial fibrosis and tubular atrophy. This pattern suggests a coherent histological profile characterized by a greater inflammatory and chronic injury burden among patients with metabolic acidosis. The absence of statistically significant differences in non-parametric testing likely reflects limited statistical power and skewed lesion distributions rather than definitive evidence of no association.

In multivariable logistic regression analyses adjusted for baseline renal function, proteinuria, and time from transplantation, metabolic acidosis was associated with higher odds of both total inflammation and IFTA; however, the confidence intervals were wide, and statistical significance was not achieved. Sensitivity analyses that additionally adjusted for hemoglobin levels and donor-related characteristics yielded directionally consistent effect estimates, indicating that the observed trends were not materially influenced by anemia status or donor factors, while highlighting the limited precision inherent in the sample size.

From a biological standpoint, the association between metabolic acidosis and histological injury is plausible. Chronic acidosis has been implicated in inflammatory activation, endothelial dysfunction, oxidative stress, and profibrotic signaling pathways, mechanisms that may contribute to microvascular inflammation and progressive structural damage in the renal allograft. Nevertheless, given the cross-sectional design of the analysis, it remains unclear whether metabolic acidosis precedes histological injury or is a downstream marker of tubular dysfunction and impaired graft function.

These findings should be interpreted as descriptive and hypothesis-generating. While they suggest that metabolic acidosis may identify a subgroup of transplant recipients with a higher histological injury burden, they do not support causal inference or clinical recommendations. Prospective longitudinal studies and interventional trials are required to clarify the temporal relationship between metabolic acidosis and histological allograft injury and to determine whether correcting acidosis can meaningfully influence graft histology or long-term outcomes.

## 5. Limitations

Several limitations of this study warrant consideration. First, the analysis was restricted to a relatively small biopsy sub-cohort from a single transplant center, which limited statistical power and widened confidence intervals. Consequently, the study may be underpowered to detect modest but clinically relevant associations, and the absence of statistical significance should be interpreted with caution.

Second, the study relied exclusively on clinically indicated kidney allograft biopsies, introducing potential indication bias. Because biopsies were performed in the setting of graft dysfunction, proteinuria, or other clinical concerns, the cohort was likely enriched for patients with underlying histological injury. This selection process may have amplified the observed coexistence of metabolic acidosis and histological lesions and limited generalizability to the broader population of stable kidney transplant recipients undergoing protocol biopsies.

Third, the cross-sectional design precludes assessment of temporality and causality. Metabolic acidosis and histological findings were assessed at a single time point, making it impossible to determine whether acidosis precedes histological injury or is a downstream marker of tubular dysfunction and graft damage. Bidirectional relationships cannot be excluded.

Fourth, although multivariable models were adjusted for key clinical confounders and supported by sensitivity analyses, residual confounding by unmeasured factors remains possible. Variables such as medication adherence, subtle or subclinical rejection, vascular health, nutritional status, and other metabolic disturbances were not fully captured and may influence both acid–base status and histological injury.

Given the exploratory nature of the analyses and the multiple histological comparisons performed, the findings should be considered hypothesis-generating rather than confirmatory. Larger, multicenter studies incorporating protocol biopsies and longitudinal follow-up are needed to validate these observations and clarify the clinical relevance of metabolic acidosis in kidney allograft histopathology.

## 6. Conclusions

In this biopsy-based, cross-sectional study, metabolic acidosis was associated with consistent directional trends toward a greater burden of both acute inflammatory and chronic histological lesions in kidney transplant recipients, although none of these associations reached statistical significance. These findings suggest that metabolic acidosis may identify a subgroup of patients with more advanced histological injury, but do not support causal inference or clinical recommendations. Larger, prospective studies incorporating protocol biopsies and interventional designs are required to clarify the temporal relationship between acid–base disturbances and allograft histopathology and to determine whether correcting metabolic acidosis influences histological injury or long-term graft outcomes.

## Figures and Tables

**Table 1:** Baseline characteristics of the biopsied cohort according to metabolic acidosis status.

Characteristics	Overall n = 63 ^1^	No Acidosis n = 22 ^1^	Metabolic Acidosis n = 41 ^1^	*p*-Value ^2^
Age, median (IQR)	43 (31, 50)	45 (38, 51)	39 (31, 49)	0.7
Sex, n (%)				0.8
Female	23 (37%)	7 (32%)	16 (39%)	
Male	40 (63%)	15 (68%)	25 (61%)	
SBP, mmHg	144 (135, 150)	148 (142, 153)	139 (131, 147)	0.08
DBP, mmHg	90 (80, 99)	93 (88, 103)	90 (80, 96)	0.10
Months from transplant, median (IQR)	24 (11, 50)	15 (8, 48)	28 (12, 50)	0.5
Donor type, n (%)				0.4
Deceased	30 (48%)	12 (55%)	18 (44%)	
Living	33 (52%)	10 (45%)	23 (56%)	
Donor age, median (IQR)	50 (43, 57)	46 (42, 55)	54 (44, 58)	0.064
Tacrolimus, n (%)	47 (74.6%)	17 (77.3%)	30 (73.2%)	1.000
Cyclosporine, n (%)	16 (25.4%)	5 (22.7%)	11 (26.8%)	1.000
Thymoglobulin induction, n (%)	6 (9.5%)	0 (0%)	6 (15%)	0.083
Basiliximab induction, n (%)	57 (90.5%)	22 (34.9%)	35 (55.6%)	0.2
Anti-HLA antibodies n (%)	37 (58.7%)	10 (45.5%)	27 (65.9%)	0.179
DGF, n (%)	4 (6.3%)	1 (4.5%)	3 (7.3%)	>0.9
eGFR, median (IQR)	50 (40, 67)	60 (49, 72)	48 (39, 62)	0.038
Creatinine, median (IQR) mg/dL	1.58 (1.31, 1.87)	1.38 (1.18, 1.76)	1.65 (1.35, 2.00)	0.082
UPCR, median (IQR) mg/g	1.10 (1.10, 1.71)	0.80 (0.80, 1.27)	1.10 (1.10, 1.78)	0.001
Hemoglobin, median IQR g/dL	12.10 (10.80, 13.50)	12.80 (11.80, 14.80)	11.20 (10.20, 13.20)	0.011
CRP, median IQR (mg/L)	4.50 (3.10, 6.70)	3.50 (2.50, 5.60)	6.00 (3.10, 7.80)	0.010
Anemia, n (%)	29 (46%)	6 (27%)	23 (56%)	0.029
Dyslipidemia, n (%)	52 (83%)	21 (95%)	31 (76%)	0.079
Obesity, n (%)	10 (16%)	2 (9.1%)	8 (20%)	0.5
Hypertension	54 (86%)	20 (91%)	34 (83%)	0.5
Charlson Comorbidity Index, n (%)				0.5
0	39 (62%)	13 (59%)	26 (63%)	
1	13 (21%)	7 (32%)	6 (15%)	
2	5 (7.9%)	1 (4.5%)	4 (9.8%)	
3	2 (3.2%)	1 (4.5%)	1 (2.4%)	
4	3 (4.8%)	0 (0%)	3 (7.3%)	
5	1 (1.6%)	0 (0%)	1 (2.4%)	

^1^ Median (Q1, Q3); n (%). ^2^ Wilcoxon rank sum test; Fisher’s exact test; Wilcoxon rank sum exact test.

**Table 2:** Distribution of acute lesion severity scores (0–3) by metabolic acidosis status.

Acidosis Status	Lesion	0	1	2	3
No acidosis	Arteritis (v)	18 (81.8%)	3 (13.6%)	1 (4.5%)	0 (0.0%)
Metabolic acidosis	Arteritis (v)	35 (85.4%)	5 (12.2%)	0 (0.0%)	1 (2.4%)
No acidosis	Glomerulitis (g)	18 (81.8%)	1 (4.5%)	0 (0.0%)	3 (13.6%)
Metabolic acidosis	Glomerulitis (g)	27 (65.9%)	9 (22.0%)	5 (12.2%)	0 (0.0%)
No acidosis	Interstitial inflammation (i)	12 (54.5%)	4 (18.2%)	3 (13.6%)	3 (13.6%)
Metabolic acidosis	Interstitial inflammation (i)	18 (43.9%)	11 (26.8%)	8 (19.5%)	4 (9.8%)
No acidosis	Peritubular capilaritis (ptc)	14 (63.6%)	6 (27.3%)	1 (4.5%)	1 (4.5%)
Metabolic acidosis	Peritubular capilaritis (ptc)	18 (43.9%)	15 (36.6%)	7 (17.1%)	1 (2.4%)
No acidosis	Tubulitis (t)	13 (59.1%)	3 (13.6%)	0 (0.0%)	6 (27.3%)
Metabolic acidosis	Tubulitis (t)	14 (34.1%)	14 (34.1%)	3 (7.3%)	10 (24.4%)

**Table 3:** Distribution of chronic lesion severity scores (0–3) by metabolic acidosis status.

Acidosis Status	Lesion	0	1	2	3
No acidosis	Vascular fibrosis intimal thickening (cv)	18 (81.8%)	2 (9.1%)	1 (4.5%)	1 (4.5%)
Metabolic acidosis	Vascular fibrosis intimal thickening (cv)	38 (92.7%)	0 (0.0%)	2 (4.9%)	1 (2.4%)
No acidosis	Glomerular basement membrane double contour (cg)	8 (36.4%)	5 (22.7%)	2 (9.1%)	7 (31.8%)
Metabolic acidosis	Glomerular basement membrane double contour (cg)	10 (24.4%)	3 (7.3%)	12 (29.3%)	16 (39.0%)
No acidosis	Arteriolar hialinosis (ah)	15 (68.2%)	5 (22.7%)	1 (4.5%)	1 (4.5%)
Metabolic acidosis	Arteriolar hialinosis (ah)	30 (73.2%)	6 (14.6%)	3 (7.3%)	2 (4.9%)
No acidosis	Interstitial fibrosis (ci)	8 (36.4%)	6 (27.3%)	5 (22.7%)	3 (13.6%)
Metabolic acidosis	Interstitial fibrosis (ci)	8 (19.5%)	17 (41.5%)	10 (24.4%)	6 (14.6%)
No acidosis	Mesangial matrix expansion (mm)	16 (72.7%)	2 (9.1%)	3 (13.6%)	1 (4.5%)
Metabolic acidosis	Mesangial matrix expansion (mm)	29 (70.7%)	5 (12.2%)	7 (17.1%)	0 (0.0%)
No acidosis	Tubular atrophy (ct)	5 (22.7%)	10 (45.5%)	3 (13.6%)	3 (13.6%)
Metabolic acidosis	Tubular atrophy (ct)	5 (12.2%)	16 (39.0%)	11 (26.8%)	9 (22.0%)

**Table 4:** Distribution of total inflammation and interstitial fibrosis/tubular atrophy (IFTA) Banff scores according to metabolic acidosis status.

Acidosis Status	Lesion	0	1	2	3
No acidosis	Total inflammation	9 (40.9%)	4 (18.4%)	6 (27.3%)	3 (13.6%)
Metabolic acidosis	Total inflammation	10 (24.4%)	10 (24.4%)	11 (26.8%)	10 (24.4%)
No acidosis	IFTA	7 (31.8%)	4 (18.2%)	5 (22.7%)	6 (27.3%)
Metabolic acidosis	IFTA	7 (17.1%)	10 (24.4%)	9 (22%)	15 (36.6%)

**Table 5:** Multivariable logistic regression analyses for the association between metabolic acidosis and histological allograft lesions.

Outcome	OR	95% CI	*p* Value
Total inflammation ≥ 1 (adjusted for eGFR + UPCRadj)	1.75	0.53–5.72	0.351
IFTA ≥ 1 (adjusted for eGFR + UPCRadj)	2.05	0.58–7.25	0.260
Total inflammation ≥ 1 (+months post-transplant)	1.78	0.53–5.86	0.342
IFTA ≥ 1 (+months post-transplant)	1.99	0.55–7.11	0.283

**Table 6:** Rejection phenotypes by metabolic acidosis status.

Variable	Overall	No Acidosis	Metabolic Acidosis	*p* Value (Fisher)
Cellular rejection	23 (36.5%)	8 (36.4%)	15 (36.6%)	1.000
Antibody-mediated rejection (ABMR)	13 (20.6%)	2 (9.1%)	11 (26.8%)	0.116

## Data Availability

The data presented in this study are not publicly available due to privacy restrictions. Upon reasonable request, the corresponding author can provide further information about the data, subject to applicable restrictions.

## Supplementary Materials

The following supporting information can be downloaded at: https://www.mdpi.com/article/10.3390/life16010097/s1, Table S1: Ordinal logistic regression analyses of individual Banff histological lesion severity according to metabolic acidosis status; Table S2: Sensitivity analyses of multivariable logistic regression models for total inflammation and IFTA.

## Abbreviations

The following abbreviations are used in this manuscript:

eGFR	Estimated Glomerular Filtration Rate
UPCR	Urinary Protein/Creatinine Ratio
DGF	Delayed Graft Function
CRP	C-Reactive Protein
SBP	Systolic Blood Pressure
DBP	Diastolic Blood Pressure
IFTA	Interstitial Fibrosis and Tubular Atrophy
