# Supplementary material for: Histological Features of Kidney Allograft Biopsies According to Metabolic Acidosis Status: A Biopsy-Based Single-Center Observational Study
Source: Life (Basel). 2026 Jan 9;16(1):97. doi: 10.3390/life16010097 (PMC12843253; doi:10.3390/life16010097)
Supplement: Supplementary file 1 [file life-16-00097-s001.zip › Supplimentary Table S1.pdf]

Supplementary Table S1. Ordinal logistic regression analyses of individual Banff histological lesion severity according to metabolic acidosis status

Histological lesion	OR for acidosis	95% CI	p-value
Glomerulitis	1.74	0.47–6.45	0.406
Peritubular capillaritis	2.48	0.83–7.48	0.106
Interstitial inflammation	1.09	0.38–3.12	0.867
Tubulitis	1.80	0.61–5.28	0.285
Arteritis	0.91	0.21–3.94	0.899
Interstitial fibrosis	1.49	0.55–4.02	0.430
Tubular atrophy	1.98	0.70–5.59	0.198
Vascular fibrosis	1.39	0.46–4.25	0.561
Total inflammation	1.75	0.65–4.74	0.272
IFTA	1.44	0.54–3.80	0.464

Ordinal logistic regression models were used to evaluate the association between metabolic acidosis and the severity of individual Banff histological lesions, treated as ordered categorical variables (scores 0–3). Odds ratios >1 indicate a higher probability of more severe lesion grades among patients with metabolic acidosis. All models were unadjusted and exploratory in nature.
