# Supplementary material for: Histological Features of Kidney Allograft Biopsies According to Metabolic Acidosis Status: A Biopsy-Based Single-Center Observational Study
Source: Life (Basel). 2026 Jan 9;16(1):97. doi: 10.3390/life16010097 (PMC12843253; doi:10.3390/life16010097)
Supplement: Supplementary file 1 [file life-16-00097-s001.zip › Supplimentary Table S2.pdf]

Supplementary Table S2. Sensitivity analyses of multivariable logistic regression models for total inflammation and IFTA

**Outcome**	**Model adjustment**	**OR (95% CI)**	**p-value**
Total inflammation ≥1	Main model (eGFR, UPCR, time from transplant)	1.87 (0.58–6.06)	0.297
Total inflammation ≥1	+ Hemoglobin	1.63 (0.49–5.46)	0.427
Total inflammation ≥1	+ Donor age and donor type	1.83 (0.55–6.07)	0.324
IFTA ≥1	Main model (eGFR, UPCR, time from transplant)	1.96 (0.56–6.81)	0.289
IFTA ≥1	+ Hemoglobin	1.62 (0.44–5.91)	0.467
IFTA ≥1	+ Donor age and donor type	1.80 (0.51–6.37)	0.361

All models were adjusted for baseline eGFR, urine protein-to-creatinine ratio (UPCR), and time from transplantation. Sensitivity analyses additionally included hemoglobin or donor-related variables, as specified.
